# Boosting teacher work engagement: the mediating role of psychological capital through emotion regulation

**DOI:** 10.3389/fpsyg.2023.1240943

**Published:** 2023-08-31

**Authors:** Yanfang Ma

**Affiliations:** College of Foreign Languages, Henan Institute of Science and Technology, Xinxiang, Henan, China

**Keywords:** EFL teachers, emotion regulation, work engagement, psychological capital, mediation model

## Abstract

**Introduction:**

This study examines the predictors of work engagement among English teachers, focusing on the mediating role of psychological capital between teacher emotion regulation and work engagement.

**Methods:**

A sample of 486 Chinese teachers participated in this research and completed self-report measures assessing emotion regulation, psychological capital, and work engagement. Structural equation modeling was employed to analyze the proposed mediation model.

**Results:**

The results revealed a positive correlation between instructor emotion regulation and both psychological capital and work engagement. Furthermore, psychological capital emerged as a significant mediator in the relationship between emotion regulation and work engagement.

**Discussion:**

The findings underscore the significance of enhancing teacher emotion regulation and psychological capital to potentially foster work engagement among educators. These results contribute to our understanding of the mechanisms that promote work engagement and have implications for the development of targeted interventions in the educational context.

## Introduction

Work engagement, conceptualized as a positive and fulfilling work-related state of mind characterized by vigor, dedication, and absorption (Schaufeli et al., 2002), has received increasing attention in organizational and occupational psychology research over the past decade. Work engagement has been found to be associated with positive outcomes such as job satisfaction, organizational commitment, and job performance (Klassen et al., 2013; Karanika-Murray et al., 2015). As a result, investigating the variables that affect work engagement is of great importance for boosting employee well-being and organizational outcomes. One group of employees who may be particularly susceptible to low levels of work engagement are teachers (Hakanen et al., 2006; Perera et al., 2018). Teaching is a demanding and challenging profession, with high levels of emotional labor and job demands that can lead to exhaustion and burnout (Huang et al., 2022). However, work engagement among teachers is critical for ensuring high-quality education and positive student outcomes (Russell and Barrett, 1999; Rothbard, 2001). As such, understanding the correlates of work engagement among teachers is essential for improving the well-being of teachers and ultimately the education of students.

Emotion regulation (ER) can be defined as the collection of techniques that individuals employ to manage their emotional states, which encompasses their moods, feelings, stress, and particular emotions (Koole, 2009; McRae and Gross, 2020). ER is especially important in second language classrooms, as instructors must continually manage their emotions owing to a variety of problems and disappointments (Zheng et al., 2022). It appears that second language (L2) instructors cannot establish a welcoming and effective learning environment in their teaching settings unless they can control their emotions, particularly their unpleasant ones (Richards, 2022; Namaziandost et al., 2023). It has been claimed that emotions are constantly altered by circumstances rather than being intrapersonal occurrences. In this sense, the instructor’s emotions can be affected by a variety of factors, ranging from their own experiences and connections with coworkers, pupils, and supervisors to the immediate cultural, political, and social settings where they are work (Smith and King, 2018).

In the last few years, there has been an upsurge in interest in positive psychology and its applicability in educational contexts (Stiglbauer et al., 2013; Carmona-Halty et al., 2021). A special focus has been placed on how pleasant emotions influence academic engagement and performance (Pekrun and Linnenbrink-Garcia, 2012; Zhang et al., 2022). Psychological capital is a positive psychological growth condition characterized by efficacy, optimism, hope, and resilience (Luthans et al., 2015). Efficacy relates to having a trusting willingness to embrace and give the required effort to complete difficult jobs. Developing a positive perception of success is referred to as optimism. Hope is defined as sticking to one’s objectives and, when required, altering one’s route in order to succeed. Lastly, when faced with obstacles and hardship, resilience means holding on, recovering, and even going above and beyond to achieve achievement (Luthans and Youssef-Morgan, 2017).

Although research has examined the relationships between teacher emotion regulation, psychological capital, and work engagement separately, few studies have explored how these variables are related to one another in a single model. The relationships among these constructs are conceptualized based on the extant literature and theoretical frameworks. It is postulated that teacher emotion regulation is directly related to work engagement, as teachers’ ability to effectively regulate emotions can impact their emotional experiences and investment in their work (Butakor et al., 2021; Greenier et al., 2021; Deng et al., 2022).

Moreover, psychological capital is hypothesized to mediate the relationship between teacher emotion regulation and work engagement (Cheung et al., 2011; Gyu Park et al., 2017). Emotionally regulated teachers may possess higher levels of self-efficacy, hope, optimism, and resilience, which, in turn, contribute to increased work engagement (Gong et al., 2019). The mediating role of psychological capital represents a theoretical mechanism through which emotion regulation is associated with work engagement, offering a comprehensive understanding of the underlying processes involved. As such, to shine new light on the relationship between these variables, the aim of this study is to explore the relationship between teacher emotion regulation and work engagement in English as a foreign language (EFL) teachers and whether psychological capital mediates this relationship. The findings of this study are of significant importance for EFL teachers, who encounter unique challenges, and further investigation into the factors contributing to work engagement among EFL teachers is highly warranted.

## Literature review

### Work engagement

Work engagement has been described from numerous angles since its inception as a relatively important construct (Bakker and Demerouti, 2008; Greenier et al., 2021; Coelho et al., 2023; Soininen et al., 2023). This consruct, according to Kahn (1990), refers to being emotionally, intellectually, and physically immersed in one’s profession. Smith et al. (2012) defined work engagement as a person’s mindset toward his or her employment, which has a direct impact on his or her psychological availability and involvement throughout role performance. According to Maslach and Leiter (2008), burnout and work engagement are two endpoints of a continuum that an employee might travel along based on his or her level of job satisfaction and participation. They used the Maslach Burnout Inventory (Maslach and Jackson, 1981) to determine an individual’s location on the burnout-engagement continuum. In contrast, Schaufeli et al. (2002) provided a definition of work engagement as a constructive state of mind that is linked to one’s job and marked by aspects of dedication, absorption, and vigor. This definition emphasizes a positive and satisfying outlook towards work and based on this concept, they then created a new scale for assessing work engagement (Schaufeli et al., 2006). As opposed to burnout, a negative idea that negatively affects one’s and others’ health and job performance, work engagement is a good aspect of employment that positively influences both people and businesses.

Work engagement is crucial in the EFL context as it directly impacts teachers’ motivation, job satisfaction, and overall effectiveness in promoting language learning and students’ academic success (Dai and Wang, 2023). Several studies have investigated factors influencing work engagement in EFL teaching, establishing significant positive relationships between self-efficacy and work engagement (Han and Wang, 2021; Xiao et al., 2022), as well as between proactive personality and flow and work engagement (Dai and Wang, 2023). Additionally, research has revealed associations between growth mindset and teaching enjoyment with work engagement and teacher grit (Liu et al., 2023), and documented the positive effects of a loving pedagogy and creativity on work engagement (Derakhshan et al., 2022). Furthermore, studies have shown positive links between reflective teaching, academic optimism, and work engagement (Li et al., 2023).

Numerous studies in the literature have explored the relationship between emotion regulation and teacher work engagement. For example, Mérida-López et al. (2017) examined a number of 288 Spanish instructors from various grade levels who worked in public schools to predict instructors’ work engagement using occupational stress and emotional intelligence indicators. The findings indicated that, first, emotion regulation was favorably related to work engagement, but uncertainty about roles and role conflict were adversely related to vigor and devotion aspects. Second, when an instructor’s experience with uncertain roles was higher, emotional intelligence was found to raise work engagement. In a similar vein, in research with 941 instructors at schools in Croatia, Burić and Macuka (2018) investigated the bidirectional relationship between instructors’ emotions and work engagement, as well as the probable influence of self-efficacy on both feelings and work engagement. The findings revealed that instructors with positive feelings had greater degrees of work engagement, whereas those with more negative emotions had lower levels of work engagement. Higher engagement levels resulted in some participants reporting fewer negative and more pleasant feelings. It was also shown that instructors with greater self-efficacy had fewer unpleasant and more favorable emotions, as well as higher work engagement. Runhaar et al. (2013) conducted two separate but related studies to investigate the impact of two professional resources, instructors’ relationships with students and their relationships with human resource practices, on instructors’ work engagement. Results of both studies revealed that positive interactions with students were associated with higher levels of work engagement, and human resources procedures were closely linked to vigor and devotion dimensions of work engagement. In a cross-cultural research, Greenier et al. (2021) showed that both individuals’ work engagement and emotion regulation were positively associated with their psychological well-being.

In another study, Deng et al. (2022) conducted an investigation into the realm of EFL teachers, uncovering a noteworthy and affirmative correlation between language teacher emotion regulation and both teachers’ self-efficacy beliefs and work engagement. Additionally, the study revealed that effective emotion regulation was associated with improved management of teachers’ anger—an important finding in the context of emotional dynamics within the teaching profession. Turning our attention to another study by Keleynikov et al. (2022), they ventured into the realm of preschool teachers to explore the potential impact of a mindfulness-based intervention aimed at nurturing compassion and emotion regulation. The outcomes were indeed remarkable, as teachers who participated in the intervention reported lower levels of emotional distress, while simultaneously exhibiting higher utilization of adaptive emotion regulation strategies, culminating in elevated levels of work engagement. These findings offer a promising avenue for promoting well-being and engagement among educators through targeted interventions.

In yet another study, Butakor et al. (2021) observed the positive influence of teachers’ emotional intelligence on both their professional identity and work engagement. The intricate connections unfolded in a dual manner, directly and indirectly through the conduit of job satisfaction. This discovery underscores the potential of emotional intelligence as a catalyst for enhancing job satisfaction and, by extension, nurturing work engagement among practicing teachers.

Collectively, these studies underscore the pivotal role of teacher emotion regulation in shaping work engagement and overall well-being. By adopting effective emotion regulation strategies, educators stand to benefit in myriad ways, with potential positive impacts on self-efficacy, job satisfaction, and professional identity—all converging to foster greater work engagement and overall teacher well-being. Consequently, investing in tailored programs that cultivate emotion regulation and emotional intelligence could prove instrumental in advancing teachers’ professional development and job satisfaction, leading to a more fulfilled and engaged teaching workforce.

### Emotion regulation

With the recent emergence of positive psychology has come a consideration of how pleasant emotions including optimism, passion, and joy can be utilized effectively in many parts of people’s lives (Seligman, 2011; Greenier et al., 2021). Emotion regulation is a notion that has been characterized in several ways since it was first proposed in the late 1990s (Cole et al., 1994). According to Thompson (1994), emotion regulation relates to both internal and external processes that an individual engages in to adjust, understand, or control their emotions to achieve desired goals. On the other hand, Cole et al. (1994) defined emotion regulation as the capacity to react to the various emotions that arise in different situations in a socially acceptable and adaptable manner. Gross (1998) described emotion regulation as a personal effort that involves multiple activities to regulate the experience and expression of emotions in terms of timing and manner.

Sutton and Harper (2009) propose that emotion regulation serves two purposes: the up-regulation of emotions to intensify feelings and the down-regulation of emotions to regulate and control specific emotional experiences. Emotion-regulation tactics are commonly utilized by instructors in the teaching profession, which is tied to recurring instances of teacher-student interactions. In this regard, instructors can either up-regulate their emotions to boost the effectiveness of instruction and effectively manage educational activities, or they can down-regulate their emotions to avoid any negative impact on pupils’ involvement, achievement, or inspiration (Gong et al., 2013). Two types of emotional regulation tactics commonly utilized by instructors were proposed by Gross (1998): response-focused strategies and antecedent-focused strategies. Instructors employ antecedent-focused strategies such as cognitive transformation, context selection, context manipulation, and attention deployment prior to the onset of emotional arousal phases. Response-focused strategies, in contrast, pertain to behavioral and physiological reactions as well as emotional expression and are engaged after the starting point of the emotional arousal phases.

The emotion regulation idea has been extensively explored, assisting in the identification of its potential associations and effects, since its inception. Katana et al. (2019), for instance, used regular diary writing to assess the 89 nurses’ well-being, perceived tension, and emotion regulation in Switzerland. According to the results of the content analysis, cognitive reappraisal increased the feeling of positive emotions, which was also favorably related with greater levels of subjective well-being and adversely associated with perceived stress. Resistance, on the other hand, which was effective for preventing the expression of negative emotions, was not substantially connected to feeling stressed or frequent well-being. Jiang et al. (2016) explored instructors’ emotions and their application of emotion regulation tools from the perspectives of the instructors and their pupils in a different investigation. The research included four Finnish professors and 53 of their pupils. Questionnaires and interviews were used to collect the necessary information. Data analysis revealed correlations between the students’ perceptions of instructors’ emotions and teachers’ views on their emotion self-regulation. Furthermore, it was found that regulating emotions after they arise (i.e., response-focused) was less effective than regulating emotions before they occur (i.e., antecedent-focused), and that changing the meaning of the situation (i.e., reappraisal) was more effective than suppressing emotions in reducing negative emotions and increasing positive emotions.

Moreover, Sutton et al. (2009) analyzed a number of publications in a review article on how instructors strive to manage the duration and strength of their emotions and how they display their feelings in the classroom. Following a review of these articles, two major results appeared. Initially, instructors tended to engage in emotion regulation in an effort to improve classroom discipline and administration, as well as their interactions with pupils. Second, instructors were more effective at transmitting good emotions to their pupils than they were at preventing negative emotions, and they used a variety of preventative and reactive emotion regulation tactics in the classroom. In another study, Ghanizadeh and Royaei (2015) investigated the dynamic relationship between emotion regulation, emotional labor methods, and instructor burnout among EFL instructors teaching at several private language institutions in Iran. According to the findings of the study, both emotion regulation and emotional labor techniques may be responsible for the feeling of teacher burnout, although negatively.

Arizmendi Tejeda et al. (2016) conducted a qualitative study to explore the strategies employed by five inexperienced EFL teachers in a southwestern Mexican city to regulate their negative emotions. The researchers collected data through semi-structured interviews and observations and used micro and constant comparative analyses to analyze the data. The results suggested that novice instructors were apprehensive in class owing to a lack of expertise in teaching and low self-esteem. When certain students did not consider them the class authority, they were dissatisfied or upset. Instructors reported using different responsive and preventative emotion-regulation tactics such as cognitive change, adjusting one’s emotional expression, choosing settings, and changing one’s emotional experience to manage such unpleasant emotions in the instructional environment. Additionally, Chahkandi et al. (2016) sought to identify the aims and tactics used by competent EFL teachers to manage their own and their pupils’ emotions. The analysis of interview data from EFL instructors as well as diary writing extracts indicated that their aims for regulating positive emotions were to demonstrate impartial instructor behavior and to retain power in the classroom, and their aims for controlling negative emotions included fostering teacher-student connections, maintaining their own and pupils’ mental health, and reinforcing instructors’ image as moral mentors.

The Broaden-and-Build theory, originally posited by Fredrickson (2013), stands as a valuable source of insights into the potential interplay between emotion regulation and work engagement. According to this theory, positive emotions play a transformative role in an individual’s cognitive and behavioral responses, enhancing their capacity for flexible and creative thinking (Fredrickson, 2013). Furthermore, positive emotions act as fundamental building blocks, nurturing the development of enduring psychological resources like resilience, optimism, and self-efficacy. Emotion regulation assumes a pivotal role in generating and sustaining these positive emotions, empowering individuals to adeptly manage negative emotional experiences while enhancing positive emotional states (Tugade and Fredrickson, 2007).

Within the context of teachers’ work engagement, proficient emotion regulation holds the potential to contribute significantly to the accumulation of psychological resources, thereby bolstering their overall well-being and levels of motivation (Sutton and Harper, 2009; De Neve et al., 2023; Gkonou and Miller, 2023). Through effective emotion regulation, teachers gain a better capacity to navigate the emotional demands intrinsic to their profession, leading to decreased emotional exhaustion and burnout (Fathi et al., 2021). As a result, teachers can preserve their invaluable psychological resources and channel them effectively into their work-related endeavors, fostering heightened levels of work engagement. Emotion regulation emerges as a critical catalyst in nurturing work engagement among teachers, as it underpins the development and continuity of positive emotional experiences and psychological resources amidst the multifaceted challenges and stressors encountered in the teaching profession (Chen et al., 2022). In essence, by cultivating adept emotion regulation, teachers can forge a path toward sustained engagement, emotional well-being, and professional fulfillment in their noble pursuit of imparting knowledge and shaping the future of their students.

The related literature has reported that emotion regulation is a significant variable affecting positive outcomes in various areas, including work engagement (Mérida-López and Extremera, 2020; George et al., 2022; Namaziandost et al., 2023). In teaching contexts, successful emotion regulation is of significant importance, since instructors might encounter a variety of emotional demands in their profession, including managing student behavior, dealing with challenging parents, and handling workload pressure (Sutton et al., 2009; Xie, 2021; Bing et al., 2022). Teachers who are better able to regulate their emotions more appropriately are more likely to experience pleasant emotions and reduce negative ones (Zhang et al., 2019; Freire et al., 2020), resulting in better job satisfaction, lower burnout, and higher levels of engagement (Viseu et al., 2016; Greenier et al., 2021).

Taken together, the role of emotion regulation in nurturing work engagement among teachers is of paramount importance. Via effectively managing their emotions, teachers gain the emotional resilience needed to navigate the challenges and demands of the ever-changing teaching profession. Developing proficient emotion regulation empowers teachers to sustain their engagement, emotional well-being, and sense of professional fulfillment, which in turn positively impacts the holistic development and growth of their students. As the field of education continues to evolve, recognizing the impact of emotion regulation on work engagement remains crucial in promoting the overall well-being of educators and fostering a nurturing and supportive learning environment for future generations.

### Psychological capital

Psychological capital is a multifaceted psychological resource consisting of efficacy, hope, optimism, and resilience (Luthans et al., 2015; Martínez et al., 2019; Preston et al., 2023). In accordance with Conservation of Resources theory (COR; Hobfoll, 2002), psychological capital (PsyCap) materials have a common theme: a positive evaluation of conditions and chance of achievement based on enthusiastic effort and tenacity (Luthans et al., 2007). In fact, according to COR, hope, efficacy, resilience, and optimism serve as “solid resource reservoir” (Hobfoll, 2002, p. 318). Stajkovic and Luthans (1998) provided a definition of efficacy as an individual’s belief or assurance regarding their capacity to activate the motivation, cognitive resources, or actions required to carry out a particular task effectively in a specific context.

In the context of education, it relates to students’ assessments of their abilities to attain their educational goals (Honicke and Broadbent, 2016; Burhanuddin et al., 2019). Despite character traits and other fixed factors of success (such as IQ or aptitude), efficacy can be learned or acquired (Bandura, 1997). Several aspects of life, involving academic performance, have demonstrated an association between efficacy and performance (Pintrich and De Groot, 1990; Zimmerman et al., 1992). Effective goal-setting is critical for generating the self-motivation, dedication, and tenacity required for academic success (Martínez et al., 2019).

In a similar vein, optimism and hope have been associated with academic achievement (Peterson and Barrett, 1987; Rand et al., 2011). As per the definition provided by Snyder et al. (1991), hope refers to a constructive incentive condition that arises from a synergistic sense of effective goal-directed action (agency) and the planning and execution of strategies to attain those goals (pathways). The desire to achieve one’s objectives is referred to as agency. Pathways are the ‘way power’ or capacity to create alternate roads to attain objectives when the original paths are obstructed by impediments (Snyder, 2000). Optimism is defined as a generally positive future perspective (i.e., anticipating favorable events to occur) along with a positive interpretive style that absorbs happy experiences while externalizing negative ones (Seligman et al., 1998; Carver et al., 2009). Optimism is essential to preserving good expectations of achievement (Martínez et al., 2019). According to Luthans (2002), resilience can be defined as the ability to recover or recuperate from hardship, disagreement, defeat, or even constructive events, advancement, and augmented accountability. In accordance with COR, resilient people maximize their social, personal, and cognitive resources, employing them effectively toward optimal adaptation behaviors and procedures with the goal of overcoming obstacles or risk factors (Masten et al., 2009).

Teacher psychological capital has gained significant attention as an essential concept that can impact several facets of a teacher’s work life, including work engagement, job satisfaction, and effectiveness as educators (Demir, 2018; Freire et al., 2020). Teacher PsyCap refers to the collection of psychological resources that teachers possess to cope with the demands and challenges of their profession (Viseu et al., 2016). These resources include their belief in their ability to teach effectively, their optimism about the future of education, their hopefulness for their students’ success, and their ability to bounce back from setbacks and adversity. Some studies have investigated the association between psychological capital and different dimensions of teacher well-being, encompassing work engagement, burnout, work satisfaction, and emotional labor. For example, in the study conducted by Joo et al. (2016) found a positive correlation between higher PsyCap and increased work engagement. The research also examined the role of work empowerment as a partial intermediary in the connection between PsyCap and work engagement, and authentic leadership as a moderator in the model. Though authentic leadership did not significantly moderate the relationship between PsyCap and work engagement, the study emphasized the significance of PsyCap in enhancing work engagement.

Also, Zhang et al. (2019) found that psychological capital acted as a protective factor against teacher burnout, while occupational stress played a risk role in contributing to burnout. Furthermore, positive coping styles emerged as substantial mediators in the relationship between psychological capital and teacher burnout, while negative coping styles mediated the link between occupational stress and burnout. In another study, Freire et al. (2020) demonstrated that flourishing partially mediated the negative impact of PsyCap on burnout symptoms. The research emphasized that both PsyCap and flourishing serve as effective personal resources in mitigating teacher burnout. Viseu et al. (2016) also highlighted the essential role of work satisfaction and positive psychological capital in motivating teachers. They called for further research to better comprehend the impact of positive psychological capital on teacher well-being and motivation. Regarding Tosten and Toprak’s (2017) study, it was observed that teachers’ PsyCap competencies influenced their emotional labor tendencies, underscoring the potential importance of psychological capital in managing emotional labor within school organizations.

Also, numerous investigations have explored the mediating role of psychological capital in diverse work settings. Gyu Park et al. (2017) uncovered that empowering leadership not only directly impacted job engagement but also indirectly influenced it through PsyCap. Moreover, PsyCap fully mediated the connection between empowering leadership and employees’ well-being, underscoring its vital role as a mechanism through which leadership behaviors can affect employee well-being. Also, Gong et al. (2019) examined the correlation between emotional intelligence (EI), job performance, and job burnout. They ascertained that psychological capital played a mediating role in these associations. Employees with higher EI displayed greater psychological capital, leading to improved job performance and decreased job burnout.

Cheung et al. (2011) found that PsyCap moderated the links between emotional labor, burnout, and job satisfaction. Specifically, high PsyCap weakened the positive association between surface acting and depersonalization, while reinforcing the positive association between deep acting and job satisfaction. Additionally, PsyCap’s relationships with depersonalization and job satisfaction were more pronounced among teachers who reported infrequent use of expression of naturally felt emotion. In another study, Roemer and Harris (2018) assessed the mediation of perceived organizational support (POS) and well-being through PsyCap. Their study established positive correlations among POS, PsyCap, and well-being. Notably, hierarchical regression analyses demonstrated PsyCap’s full mediation in the relationship between POS and well-being. Rego et al. (2016) also revealed PsyCap’s role as a mediator between authentic leadership and organizational commitment. Specifically, the dimensions of self-efficacy, hope, and optimism mediated this association, underscoring their significant impact on fostering commitment. However, the study also highlighted a negative influence of resilience on organizational commitment.

Overall, these studies underscore the significance of psychological capital in influencing multiple dimensions of teacher well-being. PsyCap plays a critical role in enhancing work engagement, reducing burnout, and improving work satisfaction among teachers. It also functions as a mechanism through which emotional intelligence, organizational support, and authentic leadership can influence work engagement, job performance, and organizational commitment. Understanding and nurturing psychological capital can contribute to establishing a positive and healthy work environment for teachers, ultimately enhancing their overall well-being and job performance.

## The hypotheses

The theoretical framework underpinning this research draws from the Conservation of Resources (COR) theory proposed by Hobfoll (1989), providing a comprehensive lens for understanding individuals’ behaviors and responses to stressors across diverse contexts, including the workplace. The COR theory posits that individuals actively strive to acquire, protect, and build resources, and the availability of these resources significantly influences their well-being, motivation, and engagement (Hobfoll, 2002). Within the context of teachers, the demands of the profession can be emotionally and cognitively taxing. Effective emotion regulation serves as a pivotal mechanism to prevent resource depletion by reducing emotional exhaustion and burnout, thus enabling teachers to conserve their valuable psychological resources (Arizmendi Tejeda et al., 2016). Consequently, this conservation process contributes to the development of higher levels of psychological capital, encompassing dimensions such as self-efficacy, optimism, hope, and resilience. Emotion regulation can thus be perceived as an active resource investment strategy, wherein teachers proficient in emotion regulation are more likely to experience positive emotions and mitigate negative emotional experiences (Sutton et al., 2009). These positive emotional encounters serve as a catalyst for the accumulation and investment in psychological capital, ultimately enhancing teachers’ well-being and fostering their work engagement.

The COR theory further postulates that resources are interconnected and can form a dynamic *resource caravan* (Hobfoll, 1989). In the context of this study, we anticipate that emotion regulation will exert a positive influence on the development of psychological capital. In turn, psychological capital, functioning as a reservoir of positive psychological resources, is expected to be related with work engagement among teachers. This interplay among resources generates a positive cycle, wherein effective emotion regulation facilitates the accumulation and mobilization of psychological capital, thereby nurturing work engagement.

Stress and negative emotions are regarded as *resource losses* within the COR theory. Through investigating how emotion regulation can ameliorate the impact of stress and negative emotions on teachers’ psychological resources (Moè and Katz, 2021), this study sheds light on the critical role of emotion regulation as a coping mechanism to preserve and enhance psychological capital (Tosten and Toprak, 2017). By understanding the intricate relationship between emotion regulation, psychological capital, and work engagement, this research contributes valuable insights into the mechanisms through which teachers can effectively manage stress and promote their well-being and engagement in the demanding context of the teaching profession. The findings can inform the development of targeted interventions and support strategies aimed at equipping teachers with the necessary emotional regulation skills to navigate the challenges of their profession and thrive in their roles. Moreover, this study aligns with the broader goals of educational research, as it explores factors that influence teacher well-being and effectiveness, ultimately contributing to the enhancement of the educational environment and student outcomes. Against this backdrop, the following hypotheses were formulated:

*H1*: Teacher emotion regulation is positively related to teacher work engagement.

The existing literature provides compelling evidence supporting the hypothesis that teacher emotion regulation is positively related to teacher work engagement. Numerous studies (Mérida-López and Extremera, 2020; Greenier et al., 2021; Chen et al., 2022; Deng et al., 2022; George et al., 2022; Namaziandost et al., 2023) have highlighted the significance of emotion regulation in influencing positive outcomes, including work engagement. In the context of teaching, where instructors encounter various emotional demands, successful emotion regulation becomes particularly crucial (Sutton et al., 2009; Xie, 2021). Teachers who effectively regulate their emotions are more likely to experience positive emotions and reduce negative ones, leading to higher job satisfaction, lower burnout, and increased work engagement (Ghanizadeh and Royaei, 2015; Katana et al., 2019; Fathi et al., 2021; Greenier et al., 2021). Effectively managing their emotions, teachers can better cope with the challenges and demands of their profession, which ultimately contributes to a more engaged and satisfied teaching experience.

*H2*: Psychological capital mediates the relationship between emotion regulation and teacher work engagement.

Recent research evidence has shown that effective emotion regulation strategies, such as reappraisal and cognitive reappraisal, positively relate to higher levels of psychological capital (Tang and He, 2022). By managing emotions adaptively, individuals are better equipped to build their resilience, maintain a positive outlook, and develop a sense of self-efficacy and hope (Mónico et al., 2016). Also, numerous studies have demonstrated a significant positive association between psychological capital and work engagement across various professional domains, including education (Joo et al., 2016; Viseu et al., 2016). Teachers with higher psychological capital are more likely to engage in their work, as their positive psychological resources drive them to be proactive, motivated, and invested in their teaching roles (Cheung et al., 2011; Viseu et al., 2016; Çimen and Ozgan, 2018). Concerning the mediating role, empirical research in occupational psychology has shown that psychological capital mediates the relationship between various individual and organizational factors and work engagement (Luthans et al., 2008; Cheung et al., 2011; Gyu Park et al., 2017; Gong et al., 2019). In this case, emotion regulation, as an individual factor, is related with work engagement indirectly through its impact on psychological capital. From this perspective, it is hypothesized that teachers who effectively regulate their emotions are more likely to develop higher psychological capital, which, in turn, fosters greater work engagement.

## Methods

### Participants

The study sample comprised 486 primary and secondary school instructors from three mainland Chinese provinces, namely Guangdong, Jiangsu, and Henan. The mean age of the participants was 36.12 (SD = 9.82), with an age range of 24 to 53 years. Of the total sample, 191 (39.21%) were male, and 295 (60.79%) were female. Among the teachers, 220 (45.3%) had been teaching for over 10 years, while 266 (54.7%) had less than 10 years of teaching experience.

The sampling procedure involved randomly selecting schools from the three provinces and sending invitations to participate in the study to the head teachers. Once a school agreed to participate, all teachers in the school were eligible to take part in the study. The sample was diverse in terms of educational levels, with 218 (44.9%) primary school teachers and 268 (55.1%) secondary school teachers. The participants’ ethnicities were primarily Han Chinese, but there were also some teachers from ethnic minority groups, including Zhuang, Hui, and Uyghur. Of the total sample, 378 (77.8%) were married, and 108 (22.2%) were single, divorced, or widowed.

### Instruments

#### Emotion regulation

To assess the emotion regulation abilities of EFL teachers, the Emotion Regulation Questionnaire developed by Gross and John (2003) was employed. This questionnaire comprises 10 items, and participants responded on a 7-point Likert-type scale, ranging from 1 (*strongly disagree*) to 7 (*strongly agree*). The questionnaire evaluates respondents’ tendencies and preferences in regulating their emotions, with a particular focus on two dimensions: Cognitive Reappraisal (CR) and Expressive Suppression (ES). An example item from the CR subscale is: “When I want to experience more positive emotions, like joy or amusement, I deliberately change my thoughts or perspective.” On the other hand, a sample item from the ES subscale is: “I tend to hide my emotions from others.”

#### Work engagement

To assess work engagement among participants, the Utrecht Work Engagement Scale (UWES), developed by Schaufeli and Bakker (2004), was employed. This scale comprises 17 items and utilizes a five-point Likert scale ranging from 1 (*strongly disagree*) to 5 (*strongly agree*). The UWES encompasses three distinct subscales, each capturing different facets of work engagement:

1. Vigour (VI) subscale measures the degree of resilience and mental strength at the job. An example item is: “At my job, I am highly resilient, both mentally and emotionally.”
2. Dedication (DE) subscale gauges the level of inspiration drawn from the job. An example item is: “My job serves as a source of inspiration and motivation.”
3. Absorption (AB) subscale assesses the extent to which individuals become fully absorbed in their work, experiencing time passing quickly. An example item is: “When I am working, time seems to fly by due to my deep engagement.”

#### Psychological capital

To assess participants’ psychological capital (PsyCap), the PCQ questionnaire developed by Luthans et al. (2015) was employed. The PCQ utilizes a Likert scale with response options ranging from 1 (*strongly disagree*) to 5 (*strongly agree*) and measures employees’ PsyCap across four key dimensions:

1. Self-efficacy (SE) dimension evaluates individuals’ belief in their ability to tackle and overcome challenges. A sample item is: “I am confident that I can effectively solve most problems if I invest the necessary effort.”
2. Hope (HO) dimension gauges individuals’ positive outlook on their future prospects. An example item is: “At present, I see myself as being quite successful.”
3. Resilience (RE) dimension assesses individuals’ capacity to bounce back and recover swiftly after facing serious life difficulties. A sample item is: “After experiencing significant life challenges, I tend to recover and adapt quickly.”
4. Optimism (OP) dimension measures individuals’ optimistic view of the life that lies ahead. An example item is: “I am looking forward to what the future has in store for me.”

The PCQ questionnaire has demonstrated satisfactory reliability and validity in previous studies (Lorenz et al., 2016; Antunes et al., 2017), making it a reliable and well-established instrument for assessing participants’ psychological capital.

### Procedure

The current research was carried out in compliance with ethical guidelines for research involving human participants, as set forth by the Institutional Review Board at the University. The study involved obtaining informed consent from the school administrators to distribute the questionnaires to Chinese primary and secondary school teachers from three provinces in mainland China, including Guangdong, Jiangsu, and Henan. A total of 600 questionnaires were distributed, and 486 were returned and deemed usable for the analysis, resulting in a return rate of 81%.

Prior to data collection, the participants were informed about the purpose of the study, which was to investigate their emotion regulation strategies, work engagement, and psychological capital. The questionnaires were distributed to the participants in two ways: either in hard copy format during regular faculty meetings or via email. The participants were given a two-week period to complete and return the questionnaires.

### Data analysis

We conducted statistical analyses using SPSS (Version 26) and Amos (Version 25) through Maximum Likelihood Estimation (MLE). To examine common method variance bias, we began by performing Harman’s single-factor test. We then used confirmatory factor analysis (CFA) to assess the measurement structure proposed by the data. Next, we utilized structural equation modelling (SEM) to evaluate the hypothesized relationships in the model. To evaluate the model’s goodness-of-fit, we used a range of indices, including Chi-square (*χ*^2^) and normed Chi-square (*χ*^2^/df), Root-Mean-Squared Error of Approximation (RMSEA), Comparative Fit Index (CFI), and Standardized Root Mean Residual (SRMR). We followed previous suggestions (Schweizer, 2010) to determine the cut-off point for model fit. Finally, we employed the bootstrap procedure with 5,000 re-samples to assess indirect effects and estimated 95% bias-corrected and accelerated (BCa) confidence intervals (CI). To further ensure the validity of our findings, we also computed McDonald’s omega reliability index, which produced comparable results to Cronbach’s alpha as recommended by Sijtsma (2009).

## Results

Before assessing the proposed model, SPSS was utilized to scrutinize the data. The examination entailed identifying missing data, assessing normality, and detecting outliers (Tabachnick and Fidell, 2007). The Expectation–Maximization (EM) algorithm was employed to manage missing data (Kline, 2011). EM is a technique of imputation that substitutes missing data with values. The normality of the items was assessed by using skewness and kurtosis indices. Values that exceeded ±2.0 indicated non-normal distribution. Lastly, both univariate and multivariate outliers were detected. The Z-Standardized scores and Mahalanobis D^2^ were used to identify univariate and multivariate outliers, respectively (Tabachnick and Fidell, 2007). This study identified and eliminated the outliers and non-normal values, resulting in the final 478 valid cases for further analysis.

Furthermore, independent samples *t*-tests revealed no significant differences in the study variables with regards to gender, age, or teaching experience. In addition, to ensure the validity of the measurement instruments in the context of the study, three separate confirmatory factor analyses (CFAs) were conducted to test the construct validity of the scales. The results indicated that the scales had adequate model fit (see Table 1).

**Table 1:** CFA results.

	CMIN	DF	CMIN/DF	*p*	CFI	RMSEA	SRMR
WE	76.086	35	2.17	0.001	0.965	0.048	0.031
PsyCap	174.846	90	1.94	0.001	0.979	0.039	0.027
ER	98.361	43	2.28	0.001	0.961	0.051	0.036

WE, work engagement; PsyCap, psychological capital; ER, emotion regulation.

The CFA results indicate that all three measurement instruments (work engagement, psychological capital, and emotion regulation) had good fit to the proposed measurement model, with CMIN/DF ratios ranging from 1.94 to 2.28 and *p* < 0.001. The CFI values were all above 0.95, indicating good fit, and the RMSEA values were all below 0.06, which is the recommended threshold for good fit. The SRMR values ranged from 0.027 to 0.036, indicating a good fit for all three instruments. Moreover, the reliability of each measurement instrument was assessed using Cronbach’s alpha coefficient. All instruments had satisfactory reliability, with alpha coefficients greater than 0.60. Additionally, McDonald’s omega reliability index was computed to cross-validate the findings and produced similar results (see Table 2).

**Table 2:** Descriptive statistics and correlations between the constructs.

Constructs	1	2	3
1. Work engagement	1		
2. Psychological capital	0.55**	1	
3. Emotion regulation	0.36**	0.46**	1
4. Mean	3.55	3.86	3.64
5. SD	0.72	0.69	0.81
6. Skewedness	−0.51	−0.22	−0.15
7. Kurtosis	−0.36	−0.63	−0.31
8. α	0.84	0.91	0.87
9. Ω	0.83	0.90	0.88

^**^p < 0.01.

Table 2 presents the descriptive statistics and correlations between the three constructs under investigation. The means and standard deviations of the constructs are also reported, along with their skewness and kurtosis indices. The table indicates that all constructs have good reliability coefficients (α > 0.84 and Ω > 0.83). The correlation coefficients show that work engagement is positively and significantly correlated with psychological capital (*r* = 0.55, *p* < 0.01) and emotion regulation (*r* = 0.36, *p* < 0.01), while psychological capital is positively and significantly correlated with emotion regulation (*r* = 0.46, *p* < 0.01). All correlations are significant at the 0.01 level.

The study then continued by examining the direct relationship between the predictor variable, emotion regulation, and the outcome variable, work engagement, through the application of SEM. This preliminary evaluation, often termed the “whole effect,” lays the foundation for subsequent mediation analysis (Little et al., 2007). Such a sequential approach is a well-established practice in SEM, ensuring the systematic exploration of the direct pathway before delving into the mediation effect, where the mediator (psychological capital) influences the initial association (Cheong and MacKinnon, 2012). Assessment of model fit was conducted employing a range of fit indices. The fit indices values were as follows: *χ*^2^/df = 1.962, CFI = 0.952, TLI = 0.947, IFI = 0.949, RMSEA = 0.048, and SRMR = 0.062. These findings demonstrated that the simplified direct model provided an adequate representation of the observed data, thereby confirming Hypothesis 1 and establishing the direct link between emotion regulation and work engagement.

**Figure 1:**
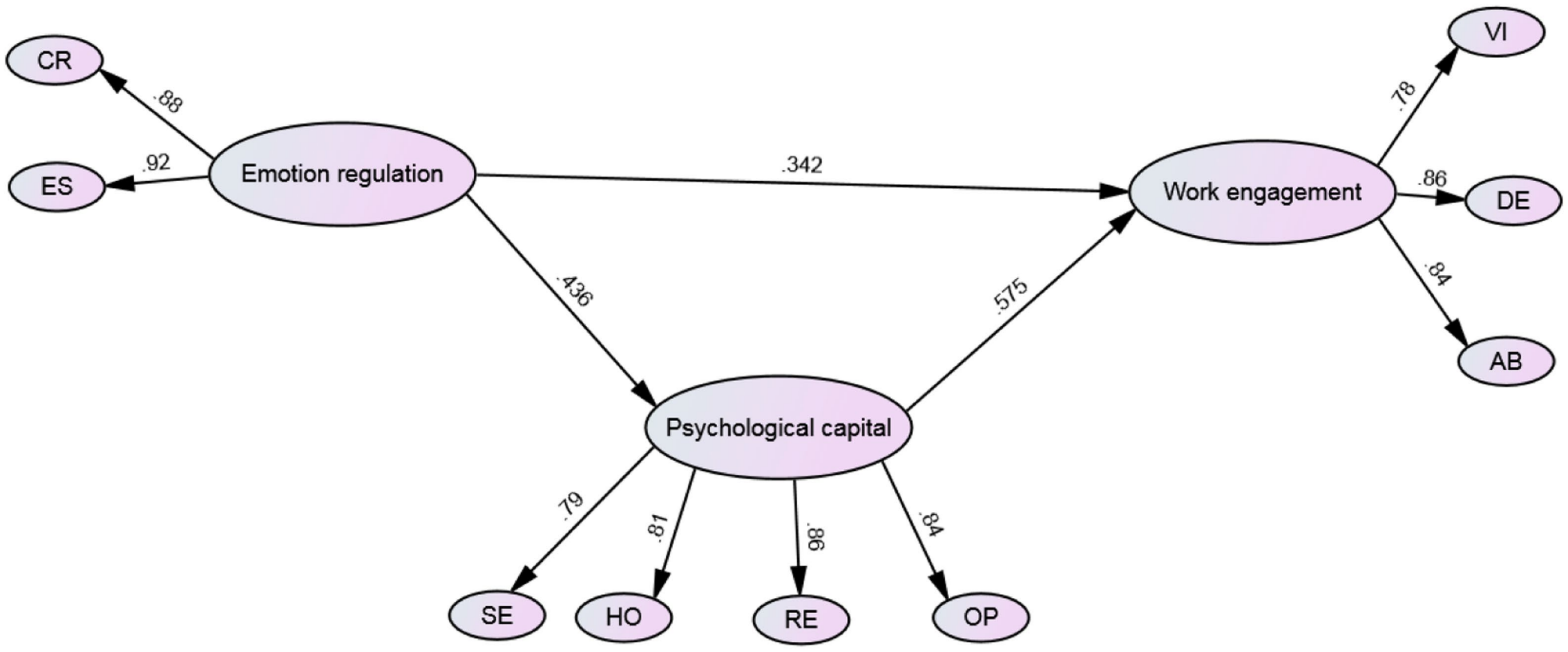
The mediation model.

With the direct relationship duly established, the study proceeded to integrate the mediator variable, psychological capital, into the model. This mediation model captures the intricate interplay among all three variables. Model fit assessment for this integrated mediation model further substantiated its robustness, with fit indices indicating a suitable fit: *χ*^2^/df = 2.080, CFI = 0.945, TLI = 0.940, IFI = 0.943, RMSEA = 0.053, and SRMR = 0.059, confirming *H2*. The standardized parameter estimates for the integrated model were visually depicted in Figure 1.

Subsequently, to rigorously evaluate the sampling distribution and indirect effects, a widely recognized SEM technique, namely 500 iterations of bootstrap resampling as introduced by Hayes (2009), was employed. This robust procedure enhances the statistical validity of assessing mediation effects and contributes to the study’s comprehensive analytical approach.

Table 3 displays the direct and indirect effects of the structural model and their respective 95% confidence intervals (CI). The *f*^2^ value, which represents the proportion of variance explained in the dependent variable by the independent variable(s), is also reported. The results indicate that emotion regulation has a significant direct effect on work engagement (*B* = 0.527, *SE* = 0.149, *β* = 0.342, *p* < 0.001), and psychological capital (*B* = 0.426, *SE* = 0.124, *β* = 0.436, *p* < 0.001). Additionally, psychological capital has a significant direct effect on work engagement (*B* = 0.767, *SE* = 0.186, *β* = 0.575, *p* < 0.001). The indirect effect of emotion regulation on work engagement via psychological capital is also significant (*B* = 0.387, *SE* = 0.076, *β* = 0.250, *p* < 0.001). The combination of teacher psychological capital and teacher emotion regulation accounted for 51.98% of the variance in work engagement, while external factors accounted for the remaining variance.

**Table 3:** Direct and indirect effects and 95% confidence intervals.

Model pathways	*B*	*SE*	*Β*	*p*	95% CI		*f* ^2^
					Lower bound	Upper bound	
*Direct effects*							
Emotion regulation→ WE	0.527	0.149	0.342	<0.001	0.129	0.524	0.189
Emotion regulation→ PsyCap	0.426	0.124	0.436	<0.001	0.394	0.682	0.392
PsyCap. → WE	0.767	0.186	0.575	<0.001	0.415	0.693	0.419
*Indirect effect*							
Emotion regulation → PsyCap → WE	0.387	0.076	0.250	<0.001	0.169	0.372	0.164

PsyCap, psychological capital; WE, work engagement.

A Harman’s single factor test was conducted to examine the possible presence of common method variance. The results revealed that the single factor explained 32.67% of the variance, which is less than the recommended threshold of 50%. Therefore, it can be concluded that common method bias was not a major concern in this study. Overall, these findings suggest that emotion regulation and psychological capital are important correlates of work engagement, and that psychological capital partially mediates the relationship between emotion regulation and work engagement.

## Discussion

The present study sought to explore the relationship between teacher emotion regulation and work engagement in Chinese EFL teachers, while also examining the mediating role of psychological capital in this dynamic. Firstly, our findings underscore a noteworthy and positive correlation between teacher emotion regulation and work engagement, aligning harmoniously with prior research (e.g., Mérida-López et al., 2017; Greenier et al., 2021; Xie, 2021; George et al., 2022; Namaziandost et al., 2023). These studies collectively emphasize the pivotal significance of emotion regulation as a predictive factor for work engagement within the realm of educators.

EFL teachers who effectively manage their emotions are better equipped to handle the emotional demands of their profession, resulting in reduced emotional exhaustion and burnout (Ghanizadeh and Royaei, 2015). Moreover, proficient emotion regulation fosters positive emotional experiences such as enthusiasm, joy, and passion, which are key drivers of work engagement (Gong et al., 2013; Butakor et al., 2021; Xie, 2021).

Furthermore, effective emotion regulation empowers teachers to navigate challenging classroom situations with composure and resilience, enabling them to maintain focus on teaching and nurturing a positive and engaging learning environment (Keleynikov et al., 2022; Dai and Wang, 2023). This reciprocal relationship forms a positive feedback loop, as higher levels of work engagement further enhance teachers’ emotion regulation abilities, leading to sustained emotional well-being and job satisfaction (Mérida-López et al., 2017; Deng et al., 2022). Beyond the confines of the classroom, engaged teachers are more proactive in seeking professional development opportunities, fostering collaboration with colleagues, and investing effort in refining their teaching practices (Cai et al., 2022; Fathi et al., 2023).

Mérida-López et al. (2017) findings emphasized that emotional intelligence remains a significant predictor of crucial elements of engagement even after accounting for factors such as instructors’ gender, age, and years of experience in the classroom. This underscores the importance of emotion regulation and psychological well-being in facilitating language instructors’ optimal performance and professional success, further accentuating the role of emotion regulation in promoting work engagement. The Broaden-and-Build theory (Fredrickson, 2013) offers a pertinent perspective, suggesting that positive emotions broaden an individual’s thought-action repertoire and build psychological resources that enable effective coping with environmental challenges. Consequently, the ability to regulate emotions plays a critical role in generating positive emotions, which, in turn, contribute to enhanced work engagement. Put simply, effective emotion regulation leads to positive emotions, broadening teachers’ thoughts and actions while simultaneously building their personal and professional resources, thereby fostering work engagement.

It can be argued that language instructors who excel in emotion regulation are more likely to be mentally, emotionally, and psychologically invested in their teaching roles. Their motivation to exert greater effort in instructional activities stems from feelings of encouragement, pride, importance, and inspiration, as they skillfully use intrinsic and external strategies to modify, evaluate, or regulate their emotions to achieve their goals. Additionally, instructors who possess better emotional control are better equipped to manage tension in the classroom, a factor that significantly impacts the work engagement of EFL teachers (Ghanizadeh and Royaei, 2015; Greenier et al., 2021), thus fostering greater commitment to their teaching duties.

The second objective of this study was to investigate the mediating role of psychological capital in the relationship between emotion regulation and work engagement among EFL teachers. The results demonstrated a significant mediating effect of psychological capital in this relationship, corroborating previous research (Cheung et al., 2011; Rego et al., 2016; Gyu Park et al., 2017; Gong et al., 2019) that highlights the pivotal role of psychological capital as a mechanism through which factors such as leadership, emotional intelligence, and organizational support influence employee well-being, work engagement, and organizational commitment.

The application of Hobfoll’s (1989) well-established Conservation of Resources (COR) theory provides a robust justification for the mediating role of psychological capital. Within the challenging landscape of the teaching profession, teachers endeavor to conserve their invaluable resources, including psychological ones, amidst the demands and pressures they face (Bettini et al., 2020). Effective emotion regulation emerges as a potent strategy enabling teachers to preserve their psychological resources by skillfully managing negative emotions and developing emotional well-being (Butakor et al., 2021). This preservation of psychological capital creates a conducive environment for heightened work engagement, as teachers are more inclined to channel their precious positive resources into their work-related pursuits (Zhang et al., 2019).

Effective emotion regulation empowers teachers to traverse a path punctuated with positive emotional experiences, mitigating emotional exhaustion, and fostering a sense of well-being (Fathi et al., 2021). These uplifting emotional encounters culminate in the development and accumulation of psychological capital, which comprises four dimensions: self-efficacy, optimism, hope, and resilience (Tosten and Toprak, 2017). Serving as an internal reservoir of positive psychological resources, psychological capital equips teachers to confront job demands and stressors (Freire et al., 2020) with resilience and a proactive spirit, thereby catalyzing their deep engagement in their work and infusing their teaching tasks with enthusiasm and dedication (Joo et al., 2016).

The empirical landscape further bolsters the association between emotion regulation strategies, such as reappraisal and cognitive reappraisal, and elevated levels of psychological capital (Tang and He, 2022). In line with existing literature (Joo et al., 2016; Roemer and Harris, 2018), our study underscores the well-established relationship between psychological capital and work engagement. Teachers with heightened psychological capital are more inclined to passionately invest themselves in their work, as their abundant positive psychological resources fuel motivation, purpose, and commitment to their teaching roles (Cheung et al., 2011; Viseu et al., 2016). These findings resonate with prior research (e.g., Bakker and Demerouti, 2007; Luthans et al., 2007), accentuating the transformative potential of psychological capital as a crucial personal resource that empowers individuals to adeptly navigate work demands and maintain unwavering levels of engagement.

Lastly, the non-significant differences in emotion regulation and work engagement across gender, age, and teaching experience suggest that these constructs may have universal relevance and applicability to all teachers (Ford and Lerner, 1992). It also indicates that teachers, irrespective of gender, age, or teaching experience, are adept at employing effective emotion regulation strategies and maintaining positive psychological resources to sustain their work engagement. This finding aligns with theoretical models that emphasize the universality of certain psychological constructs, such as the COR theory, which posits that individuals endeavor to conserve and build resources across diverse contexts. The absence of significant differences in this study lends support to the notion that emotion regulation and work engagement are integral components of a general resource preservation and accumulation process, less influenced by demographic factors.

## Conclusions and implications

The current study intended to explore the relationship between teacher emotion regulation and work engagement in EFL teachers and whether psychological capital mediated this relationship. According to the findings of this study, there was a positive relationship between teacher emotion regulation and work engagement in EFL teachers. Language teachers who are more proficient at managing their emotions are more likely to develop a mental, emotional, and psychological attachment to their teachings; they also feel satisfied, motivated, and become totally and passionately engaged in their professional role. It was also found that there was a positive relationship between psychological capital and work engagement among EFL teachers. People can have prosperous and meaningful lives by having psychological capital, which acts as a beneficial guiding mindset. The reason for this is that people who have access to psychological resources, like PsyCap, are more joyful and more engaged at work.

The findings unveil a wealth of promising implications, both theoretically and practically, that contribute novel insights to the realm of work engagement. Theoretically, our research enriches the existing literature on work engagement by illuminating the intricate mediating role of psychological capital in the dynamic relationship between teacher emotion regulation and work engagement. This discovery resonates harmoniously with prior studies, affirming the paramount importance of psychological capital as a potent resource capable of buffering against stress and fostering well-being within the workplace (Luthans and Youssef-Morgan, 2017). By unraveling the mediating effect of psychological capital, our study unveils the hidden mechanisms that propel the connection between emotion regulation and work engagement, adding depth and nuance to the understanding of English teachers’ experiences.

Practically, our findings bear transformative implications for educators and school administrators, paving the way for targeted interventions that can enhance teachers’ work engagement and overall welfare. Professional development programs geared towards teachers should transcend the conventional focus on instructional skills and acknowledge the pivotal role of emotion regulation strategies. Equipping teachers with robust training and unwavering support in effectively managing their emotions bestows them with heightened psychological capital, fostering a harmonious and invigorating work environment. With enlightened school administrators at the helm, policies and practices that champion emotion regulation and psychological capital development can take root, developing a supportive and inclusive school culture that cherishes teachers’ emotional well-being, thereby catalyzing their work engagement and job satisfaction. Integrating modules on emotion regulation and psychological capital within teacher training programs will illuminate their relevance and profound impact on work engagement, empowering future educators to adeptly navigate the emotional challenges intrinsic to the teaching profession, perpetuating sustained engagement and unwavering resilience throughout their careers. Moreover, our findings shed light on the pertinence of recognizing individual differences in emotion regulation and psychological capital among teachers. Embracing a tailored approach, school leaders and administrators can adeptly cater to the unique needs of each teacher, thereby fostering a utopian work environment characterized by unwavering positivity and thriving spirits.

As an example, educators, principals, school psychologists, and therapists are urged to collaborate on developing, preparing, and implementing educational and therapy programs that seek to develop pupils. Long-term psychological capabilities, such as psychological capital, to support beneficial mental health in educational settings. Implementing PsyCap-oriented courses could not only improve teachers’ wellbeing and work engagement, but also stop the occurrence of unfavorable outcomes (such as anxiety, burnout, and quitting the job). The acquired data can help educational administrators at universities focus on psycho-emotional dimensions, including ER among university instructors. For instance, they can conduct exams to see if university instructors can effectively control their emotions, and if not, they can schedule therapeutic psychological consultations to address the issue. The results may also help teachers include ER in their curriculum so that teaching students become familiar with it for their careers. In the end, the findings may help university instructors understand that they must control their feelings at work if they want to increase their confidence, workplace-derangement, and rage.

Moreover, the study sheds light on the unique challenges faced by EFL teachers and stresses the importance of addressing these challenges in interventions aimed at enhancing their work engagement. Language barriers, cultural differences, and other challenges in teaching should be considered when designing interventions that help EFL teachers stay engaged in their work. Finally, the findings might underscore the necessity for cross-cultural research to explore the generalizability of the findings to different cultural contexts. Such research could help identify cultural differences that need to be considered when designing interventions aimed at enhancing work engagement among EFL teachers in different settings.

Despite the profound implications of our discoveries, it is vital to acknowledge the following limitations, which call for further exploration and refinement. Firstly, data collection relied on self-report measures, possibly susceptible to common method bias and the covert influence of social desirability. Respondents might have leaned towards socially acceptable responses, potentially skewing or inflating the outcomes. To fortify the validity of our findings, future investigations could integrate diverse data sources, such as objective performance measures or observer ratings, to gain a more comprehensive understanding.

Secondly, the cross-sectional nature of our study restricts causal inferences between the variables under scrutiny. While we tested the proposed mediation model, the true directionality of relationships remains elusive. To bolster the evidential foundation for the mediating role of psychological capital in the link between emotion regulation and work engagement, longitudinal or experimental research designs may offer more compelling evidence. Furthermore, our sample exclusively comprised Chinese teachers, which may hinder generalizing the findings to educators from distinct cultural backgrounds or educational systems. Culture and contextual factors can wield a substantial influence on emotion regulation and work engagement. Replicating the study in diverse cultural settings is essential to assess the robustness and cross-cultural applicability of the results.

Moreover, our study predominantly relied on quantitative measures, overlooking the valuable insights qualitative data can offer into the lived experiences of teachers concerning emotion regulation and work engagement. Integrating qualitative research methods, such as interviews or focus groups, can unlock a richer understanding of the underlying mechanisms that drive the observed relationships. Lastly, we did not account for potential confounding variables that might exert an influence on the relationship between emotion regulation, psychological capital, and work engagement. Variables like job demands, organizational support, and individual traits could interact with the studied constructs, potentially affecting the outcomes. Future studies could incorporate additional control variables to refine the precision of the findings and enhance the overall comprehensiveness of the research.

## Data availability statement

The original contributions presented in the study are included in the article/supplementary material, further inquiries can be directed to the corresponding author.

## Ethics statement

The studies involving humans were approved by College of Foreign Languages, Henan Institute of Science and Technology, Xinxiang, Henan, China. The studies were conducted in accordance with the local legislation and institutional requirements. The participants provided their written informed consent to participate in this study.

## Author contributions

The author confirms being the sole contributor of this work and has approved it for publication.

## Conflict of interest

The author declares that the research was conducted in the absence of any commercial or financial relationships that could be construed as a potential conflict of interest.

## Publisher’s note

All claims expressed in this article are solely those of the authors and do not necessarily represent those of their affiliated organizations, or those of the publisher, the editors and the reviewers. Any product that may be evaluated in this article, or claim that may be made by its manufacturer, is not guaranteed or endorsed by the publisher.

## References

[ref1] Arizmendi Tejeda S. Gillings de González B. S. López Martínez C. L. D. J. (2016). How novice EFL teachers regulate their negative emotions. How 23, 30–48. doi: 10.19183/how.23.1.299

[ref002] Antunes A. C. Caetano A. Pina e Cunha M. (2017). Reliability and construct validity of the Portuguese version of the Psychological Capital Questionnaire. Psychol. Rep 120, 520–536. doi: 10.1177/0033294116686742 28558609

[ref2] Bakker A. B. Demerouti E. (2007). The job demands-resources model: state of the art. J. Manag. Psychol. 22, 309–328. doi: 10.1108/02683940710733115

[ref3] Bakker A. B. Demerouti E. (2008). Towards a model of work engagement. Career Dev. Int. 13, 209–223. doi: 10.1108/13620430810870476

[ref4] Bandura A. (1997). Self-Efficacy: The Exercise of Control. New York: Freeman.

[ref5] Bettini E. Gilmour A. F. Williams T. O. Billingsley B. (2020). Predicting special and general educators’ intent to continue teaching using conservation of resources theory. Except. Child. 86, 310–329. doi: 10.1177/0014402919870464

[ref6] Bing H. Sadjadi B. Afzali M. Fathi J. (2022). Self-efficacy and emotion regulation as predictors of teacher burnout among English as a foreign language teachers: a structural equation modeling approach. Front. Psychol. 13:900417. doi: 10.3389/fpsyg.2022.900417, PMID: 35664188 PMC9160989

[ref7] Burhanuddin N. A. N. Ahmad N. A. Said R. R. Asimiran S. (2019). A systematic review of the psychological capital (PsyCap) research development: implementation and gaps. Int. J. Acad. Res. Prog. Educ. Dev. 8, 133–150. doi: 10.6007/IJARPED/v8-i3/6304

[ref8] Burić I. Macuka I. (2018). Self-efficacy, emotions and work engagement among teachers: a two wave cross-lagged analysis. J. Happiness Stud. 19, 1917–1933. doi: 10.1007/s10902-017-9903-9

[ref9] Butakor P. K. Guo Q. Adebanji A. O. (2021). Using structural equation modeling to examine the relationship between Ghanaian teachers' emotional intelligence, job satisfaction, professional identity, and work engagement. Psychol. Sch. 58, 534–552. doi: 10.1002/pits.22462

[ref10] Cai Y. Wang L. Bi Y. Tang R. (2022). How can the professional community influence teachers’ work engagement? The mediating role of teacher self-efficacy. Sustainability 14:10029. doi: 10.3390/su141610029

[ref11] Carmona-Halty M. Salanova M. Llorens S. Schaufeli W. B. (2021). Linking positive emotions and academic performance: the mediated role of academic psychological capital and academic engagement. Curr. Psychol. 40, 2938–2947. doi: 10.1007/s12144-019-00227-8

[ref12] Carver C. S. Scheier M. F. Miller C. J. Fulford D. (2009). “Optimism” in Oxford Handbook of Positive Psychology. eds. Lopez S. J. Snyder C. R.. 2nd ed (Oxford: Oxford University Press), 303–311.

[ref13] Chahkandi F. Rasekh A. E. Tavakoli M. (2016). Efficacious EFL teachers’ goals and strategies for emotion management: the role of culture in focus. Iran. J. Appl. Linguist. 19, 35–72. doi: 10.18869/acadpub.ijal.19.1.35

[ref14] Chen L. Li X. Xing L. (2022). From mindfulness to work engagement: the mediating roles of work meaningfulness, emotion regulation, and job competence. Front. Psychol. 13:6701. doi: 10.3389/fpsyg.2022.997638, PMID: 36389549 PMC9643705

[ref15] Cheong J. W. MacKinnon D. (2012). “Mediation/indirect effects in structural equation modeling” in Handbook of Structural Equation Modeling. ed. Hoyle R. H. (London: Guilford Press), 417–435.

[ref16] Cheung F. Tang C. S. K. Tang S. (2011). Psychological capital as a moderator between emotional labor, burnout, and job satisfaction among school teachers in China. Int. J. Stress. Manag. 18, 348–371. doi: 10.1037/a0025787

[ref17] Çimen İ. Ozgan H. (2018). Contributing and damaging factors related to the psychological capital of teachers: a qualitative analysis. Issues Educ. Res. 28, 308–328.

[ref18] Coelho G. L. D. H. da Fonsêca P. N. Vilar R. de Carvalho Mendes L. A. Gouveia V. V. (2023). How can human values influence work engagement among teachers? An exploratory study. Trends Psychol., 1–14. doi: 10.1007/s43076-023-00258-y PMC9908304 40479490

[ref19] Cole P. M. Michel M. K. Teti L. O. D. (1994). The development of emotion regulation and dysregulation: a clinical perspective. Monogr. Soc. Res. Child Dev. 59, 73–102. doi: 10.2307/1166139, PMID: 7984169

[ref20] Dai K. Wang Y. (2023). Investigating the interplay of Chinese EFL teachers’ proactive personality, flow, and work engagement. J. Multiling. Multicult. Dev., 1–15. doi: 10.1080/01434632.2023.2174128

[ref21] De Neve D. Bronstein M. V. Leroy A. Truyts A. Everaert J. (2023). Emotion regulation in the classroom: a network approach to model relations among emotion regulation difficulties, engagement to learn, and relationships with peers and teachers. J. Youth Adolesc. 52, 273–286. doi: 10.1007/s10964-022-01678-2, PMID: 36180661 PMC9524346

[ref22] Demir S. (2018). The relationship between psychological capital and stress, anxiety, burnout, job satisfaction, and job involvement. Eurasian J. Educ. Res. 75, 137–153.

[ref23] Deng J. Heydarnejad T. Farhangi F. Farid Khafaga A. (2022). Delving into the relationship between teacher emotion regulation, self-efficacy, engagement, and anger: a focus on English as a foreign language teachers. Front. Psychol. 13:1019984. doi: 10.3389/fpsyg.2022.1019984, PMID: 36337515 PMC9627275

[ref24] Derakhshan A. Greenier V. Fathi J. (2022). Exploring the interplay between a loving pedagogy, creativity, and work engagement among EFL/ESL teachers: a multinational study. Curr. Psychol., 1–20. doi: 10.1007/s12144-022-03371-w

[ref25] Fathi J. Greenier V. Derakhshan A. (2021). Self-efficacy, reflection, and burnout among Iranian EFL teachers: the mediating role of emotion regulation. Iranian journal of language teaching. Research 9, 13–37. doi: 10.30466/IJLTR.2021.121043

[ref26] Fathi J. Zhang L. J. Arefian M. H. (2023). Testing a model of EFL teachers’ work engagement: the roles of teachers’ professional identity, L2 grit, and foreign language teaching enjoyment. Int. Rev. Appl. Linguist. Lang. Teach. doi: 10.1515/iral-2023-0024

[ref27] Ford D. H. Lerner R. M. (1992). Developmental Systems Theory: An Integrative Approach Sage Publications, Inc.

[ref28] Fredrickson B. L. (2013). “Positive emotions broaden and build” in Advances in Experimental Social Psychology, vol. 47 (Academic Press), 1–53.

[ref29] Freire C. Ferradás M. D. M. García-Bértoa A. Núñez J. C. Rodríguez S. Piñeiro I. (2020). Psychological capital and burnout in teachers: the mediating role of flourishing. Int. J. Environ. Res. Public Health 17:8403. doi: 10.3390/ijerph17228403, PMID: 33202826 PMC7697347

[ref30] George O. J. Okon S. E. Akaighe G. (2022). Emotional intelligence and work engagement: a serial mediation model. J. Organ. Eff. People Perform 9, 193–211. doi: 10.1108/JOEPP-02-2021-0025

[ref31] Ghanizadeh A. Royaei N. (2015). Emotional facet of language teaching: emotion regulation and emotional labor strategies as predictors of teacher burnout. Int. J. Pedagogies Learn. 10, 139–150. doi: 10.1080/22040552.2015.1113847

[ref32] Gkonou C. Miller E. R. (2023). Relationality in language teacher emotion regulation: regulating emotions through, with and for others. System 115:103046. doi: 10.1016/j.system.2023.103046

[ref33] Gong S. Chai X. Duan T. Zhong L. Jiao Y. (2013). Chinese teachers’ emotion regulation goals and strategies. Psychology 4, 870–877. doi: 10.4236/psych.2013.411125

[ref34] Gong Z. Chen Y. Wang Y. (2019). The influence of emotional intelligence on job burnout and job performance: mediating effect of psychological capital. Front. Psychol. 10:2707. doi: 10.3389/fpsyg.2019.02707, PMID: 31920783 PMC6916327

[ref35] Greenier V. Derakhshan A. Fathi J. (2021). Emotion regulation and psychological well-being in teacher work engagement: a case of British and Iranian English language teachers. System 97:102446. doi: 10.1016/j.system.2020.102446

[ref36] Gross J. J. (1998). The emerging field of emotion regulation: an integrative review. Rev. Gen. Psychol. 2, 271–299. doi: 10.1037/1089-2680.2.3.271

[ref37] Gross J. J. John O. P. (2003). Individual differences in two emotion regulation processes: implications for affect, relationships, and well-being. J. Pers. Soc. Psychol. 85, 348–362. doi: 10.1037/0022-3514.85.2.348, PMID: 12916575

[ref38] Gyu Park J. Sik Kim J. Yoon S. W. Joo B. K. (2017). The effects of empowering leadership on psychological well-being and job engagement: the mediating role of psychological capital. Leadersh. Organ. Dev. J. 38, 350–367. doi: 10.1108/LODJ-08-2015-0182

[ref39] Hakanen J. J. Bakker A. B. Schaufeli W. B. (2006). Burnout and work engagement among teachers. J. Sch. Psychol. 43, 495–513. doi: 10.1016/j.jsp.2005.11.001

[ref40] Han Y. Wang Y. (2021). Investigating the correlation among Chinese EFL teachers' self-efficacy, work engagement, and reflection. Front. Psychol. 12:763234. doi: 10.3389/fpsyg.2021.763234, PMID: 34803845 PMC8603392

[ref41] Hayes A. F. (2009). Beyond baron and Kenny: statistical mediation analysis in the new millennium. Commun. Monogr. 76, 408–420. doi: 10.1080/03637750903310360

[ref42] Hobfoll S. E. (1989). Conservation of resources: a new attempt at conceptualizing stress. Am. Psychol. 44, 513–524. doi: 10.1037/0003-066X.44.3.513, PMID: 2648906

[ref43] Hobfoll S. E. (2002). Social and psychological resources and adaptation. Rev. Gen. Psychol. 6, 307–324. doi: 10.1037/1089-2680.6.4.307

[ref44] Honicke T. Broadbent J. (2016). The influence of academic self-efficacy on academic performance: a systematic review. Educ. Res. Rev. 17, 63–84. doi: 10.1016/j.edurev.2015.11.002

[ref45] Huang S. Y. Huang C. H. Chang T. W. (2022). A new concept of work engagement theory in cognitive engagement, emotional engagement, and physical engagement. Front. Psychol. 12:6503. doi: 10.3389/fpsyg.2021.663440, PMID: 35242067 PMC8886307

[ref46] Jiang J. Vauras M. Volet S. Wang Y. (2016). Teachers' emotions and emotion regulation strategies: self-and students' perceptions. Teach. Teach. Educ. 54, 22–31. doi: 10.1016/j.tate.2015.11.008

[ref47] Joo B. K. Lim D. H. Kim S. (2016). Enhancing work engagement: the roles of psychological capital, authentic leadership, and work empowerment. Leadersh. Organ. Dev. J. 37, 1117–1134. doi: 10.1108/LODJ-01-2015-0005

[ref001] Kahn W. A. (1990). Psychological conditions of employee engagement and disengagement at work. Acad. Manage. J. 33, 692–724. doi: 10.5465/256287

[ref48] Karanika-Murray M. Duncan N. Pontes H. M. Griffiths M. D. (2015). Organizational identification, work engagement, and job satisfaction. J. Manag. Psychol. 30, 1019–1033. doi: 10.1108/JMP-11-2013-0359

[ref49] Katana M. Röcke C. Spain S. M. Allemand M. (2019). Emotion regulation, subjective well-being, and perceived stress in daily life of geriatric nurses. Front. Psychol. 10:1097. doi: 10.3389/fpsyg.2019.01097, PMID: 31156513 PMC6529805

[ref50] Keleynikov M. Benatov J. Berger R. (2022). Preschool teachers’ psychological distress and work engagement during COVID-19 outbreak: the protective role of mindfulness and emotion regulation. Int. J. Environ. Res. Public Health 19:2645. doi: 10.3390/ijerph19052645, PMID: 35270334 PMC8909723

[ref51] Klassen R. M. Yerdelen S. Durksen T. L. (2013). Measuring teacher engagement: development of the engaged teachers scale (ETS). Frontline Learn. Res. 1, 33–52. doi: 10.14786/flr.v1i2.44

[ref52] Kline R. B. (2011). Principles and Practice of Structural Equation Modeling. 3nd. New York: Guilford Press.

[ref53] Koole S. L. (2009). The psychology of emotion regulation: an integrative review. Cognit. Emot. 23, 4–41. doi: 10.1080/02699930802619031

[ref54] Li F. Mohammaddokht F. Hosseini H. M. Fathi J. (2023). Reflective teaching and academic optimism as correlates of work engagement among university instructors. Heliyon 9:e13735. doi: 10.1016/j.heliyon.2023.e13735, PMID: 36865456 PMC9971167

[ref55] Little T. D. Card N. A. Bovaird J. A. Kristopher J. P. Crandall C. S. (2007). “Structural equation modeling of mediation and moderation with contextual factors” in Modeling Contextual Effects in Longitudinal Studies. eds. Bovaird J. A. Little T. D. Card N. A. (New York: Routledge), 207–230.

[ref56] Liu L. Fathi J. Allahveysi S. P. Kamran K. (2023). A model of teachers’ growth mindset, teaching enjoyment, work engagement, and teacher grit among EFL teachers. Front. Psychol. 14:1137357. doi: 10.3389/fpsyg.2023.1137357, PMID: 36968701 PMC10030517

[ref003] Lorenz T. Beer C. Pütz J. Heinitz K. (2016). Measuring psychological capital: Construction and validation of the compound PsyCap scale (CPC-12). PLoS One. 11, 1–17. doi: 10.1371/journal.pone.0152892 PMC4817957 27035437

[ref57] Luthans F. (2002). Positive organizational behavior: developing and managing psychological strengths. Acad. Manag. Perspect. 16, 57–72. doi: 10.5465/ame.2002.6640181

[ref58] Luthans F. Avolio B. J. Avey J. B. Norman S. M. (2007). Positive psychological capital: measurement and relationship with performance and satisfaction. Pers. Psychol. 60, 541–572. doi: 10.1111/j.1744-6570.2007.00083.x

[ref59] Luthans B. C. Luthans K. W. Jensen S. M. (2012). The impact of business school students’ psychological capital on academic performance. J. Educ. Bus. 87, 253–259. doi: 10.1080/08832323.2011.609844

[ref60] Luthans F. Norman S. M. Avolio B. J. Avey J. B. (2008). The mediating role of psychological capital in the supportive organizational climate—employee performance relationship. J. Organ. Behav. 29, 219–238. doi: 10.1002/job.507

[ref61] Luthans F. Youssef C. M. Avolio B. J. (2015). Psychological Capital and Beyond. Oxford University Press, USA.

[ref62] Luthans F. Youssef-Morgan C. M. (2017). Psychological capital: an evidence-based positive approach. Annu. Rev. Organ. Psych. Organ. Behav. 4, 339–366. doi: 10.1146/annurev-orgpsych-032516-113324

[ref63] Martínez I. M. Youssef-Morgan C. M. Chambel M. J. Marques-Pinto A. (2019). Antecedents of academic performance of university students: academic engagement and psychological capital resources. Educ. Psychol. 39, 1047–1067. doi: 10.1080/01443410.2019.1623382

[ref64] Maslach C. Jackson S. E. (1981). The measurement of experienced burnout. J. Organ. Behav. 2, 99–113. doi: 10.1002/job.4030020205

[ref65] Maslach C. Leiter M. P. (2008). The Truth about Burnout: How Organizations Cause Personal Stress and What to Do About It John Wiley & Sons.

[ref66] Masten A. S. Cutuli J. J. Herbers J. E. Reed M. G. J. (2009). “Resilience in development” in Oxford Handbook of Positive Psychology. eds. Lopez S. J. Snyder C. R.. 2nd ed (New York: Oxford University Press), 117–131.

[ref67] McIntyre T. McIntyre S. Francis D. (2017). Educator Stress. New York, NY: Springer, 10, 978–973.

[ref68] McRae K. Gross J. J. (2020). Emotion regulation. Emotion 20, 1–9. doi: 10.1037/emo0000703 31961170

[ref69] Mérida-López S. Extremera N. (2020). The interplay of emotional intelligence abilities and work engagement on job and life satisfaction: which emotional abilities matter most for secondary-school teachers? Front. Psychol. 11:563634. doi: 10.3389/fpsyg.2020.563634, PMID: 33192836 PMC7606868

[ref70] Mérida-López S. Extremera N. Rey L. (2017). Contributions of work-related stress and emotional intelligence to teacher engagement: additive and interactive effects. Int. J. Environ. Res. Public Health 14:1156. doi: 10.3390/ijerph14101156, PMID: 28961218 PMC5664657

[ref71] Moè A. Katz I. (2021). Emotion regulation and need satisfaction shape a motivating teaching style. Teach. Teach. 27, 370–387. doi: 10.1080/13540602.2020.1777960

[ref72] Mónico L. Mellão N. Nobre-Lima L. Parreira P. Carvalho C. (2016). Emotional intelligence and psychological capital: what is the role of workplace spirituality. Rev. Port. Enferm. Saúde Mental 3, 45–50. doi: 10.19131/rpesm.0116

[ref73] Namaziandost E. Heydarnejad T. Rahmani Doqaruni V. Azizi Z. (2023). Modeling the contributions of EFL university professors’ emotion regulation to self-efficacy, work engagement, and anger. Curr. Psychol. 42, 2279–2293. doi: 10.1007/s12144-022-04041-7, PMID: 36531192 PMC9735219

[ref74] Pekrun R. Linnenbrink-Garcia L. (2012). “Academic emotions and student engagement” in Handbook of Research on Student Engagement, 259–282.

[ref75] Perera H. N. Granziera H. McIlveen P. (2018). Profiles of teacher personality and relations with teacher self-efficacy, work engagement, and job satisfaction. Personal. Individ. Differ. 120, 171–178. doi: 10.1016/j.paid.2017.08.034

[ref76] Peterson C. Barrett L. C. (1987). Explanatory style and academic performance among university freshman. J. Pers. Soc. Psychol. 53, 603–607. doi: 10.1037/0022-3514.53.3.603

[ref77] Pintrich P. R. De Groot E. V. (1990). Motivational and self-regulated learning components of classroom academic performance. J. Educ. Psychol. 82, 33–40. doi: 10.1037/0022-0663.82.1.33

[ref78] Preston A. Rew L. Young C. C. (2023). A systematic scoping review of psychological capital related to mental health in youth. J. Sch. Nurs. 39, 72–86. doi: 10.1177/10598405211060415, PMID: 34898323

[ref79] Rand K. L. Martin A. D. Shea A. M. (2011). Hope, but not optimism, predicts academic performance of law students beyond previous academic achievement. J. Res. Pers. 45, 683–686. doi: 10.1016/j.jrp.2011.08.004

[ref80] Rego P. Lopes M. P. Nascimento J. L. (2016). Authentic leadership and organizational commitment: the mediating role of positive psychological capital. J. Ind. Eng. Manage. 9, 129–151. doi: 10.3926/jiem.1540

[ref81] Richards J. C. (2022). Exploring emotions in language teaching. RELC J. 53, 225–239. doi: 10.1177/0033688220927531

[ref82] Roemer A. Harris C. (2018). Perceived organisational support and well-being: the role of psychological capital as a mediator. SA J. Ind. Psychol. 44, 1–11. doi: 10.4102/sajip.v44i0.1539

[ref83] Rothbard N. P. (2001). Enriching or depleting? The dynamics of engagement in work and family roles. Adm. Sci. Q. 46, 655–684. doi: 10.2307/3094827

[ref84] Runhaar P. Konermann J. Sanders K. (2013). Teachers' organizational citizenship behaviour: considering the roles of their work engagement, autonomy and leader–member exchange. Teach. Teach. Educ. 30, 99–108. doi: 10.1016/j.tate.2012.10.008

[ref85] Russell J. A. Barrett L. F. (1999). Core affect, prototypical emotional episodes, and other things called emotion: dissecting the elephant. J. Pers. Soc. Psychol. 76, 805–819. doi: 10.1037/0022-3514.76.5.805, PMID: 10353204

[ref86] Schaufeli W. Bakker A. (2004). Utrecth Work Engagement Scale. Preliminary Manual. Utrecht: Utrech University.

[ref87] Schaufeli W. B. Bakker A. B. Salanova M. (2006). The measurement of work engagement with a short questionnaire: a cross-national study. Educ. Psychol. Meas. 66, 701–716. doi: 10.1177/0013164405282471

[ref88] Schaufeli W. B. Salanova M. González-Romá V. Bakker A. B. (2002). The measurement of engagement and burnout: a two sample confirmatory factor analytic approach. J. Happiness Stud. 3, 71–92. doi: 10.1023/A:1015630930326

[ref89] Schweizer K. (2010). Some guidelines concerning the modelling of traits and abilities in test construction. Eur. J. Psychol. Assess. 26, 1–2. doi: 10.1027/1015-5759/a000001

[ref90] Seligman M. E. (2011). Flourish: A Visionary New Understanding of Happiness and Well-Being Simon and Schuster.

[ref91] Seligman M. E. P. Abramson L. Y. Semmel A. Von Bayer C. (1998). Learned Optimism New York Pocket Books.

[ref92] Sijtsma K. (2009). On the use, the misuse, and the very limited useful- ness of Cronbach’s alpha. Psychometrika 74, 107–120. doi: 10.1007/s11336-008-9101-0 20037639 PMC2792363

[ref93] Smith L. King J. (2018). “Silence in the foreign language classroom: the emotional challenges for L2 teachers” in Emotions in Second Language Teaching: Theory, Research and Teacher Education. ed. Martínez Agudo J. D. D. (Switzerland: Springer), 323–339.

[ref94] Smith M. R. Rasmussen J. L. Mills M. J. Wefald A. J. Downey R. G. (2012). Stress and performance: do service orientation and emotional energy moderate the relationship? J. Occup. Health Psychol. 17, 116–128. doi: 10.1037/a0026064, PMID: 22122550

[ref95] Snyder C. R. (2000). Handbook of Hope. San Diego: Academic Press.

[ref96] Snyder C. R. Harris C. Anderson J. R. Holleran S. A. Irving L. M. Sigmon S. T.. (1991). The will and the ways: development and validation of an individual-differences measure of hope. J. Pers. Soc. Psychol. 60, 570–585. doi: 10.1037/0022-3514.60.4.570, PMID: 2037968

[ref97] Soininen V. Pakarinen E. Lerkkanen M. K. (2023). Reciprocal associations among teacher–child interactions, teachers' work engagement, and children's social competence. J. Appl. Dev. Psychol. 85:101508. doi: 10.1016/j.appdev.2022.101508

[ref98] Stajkovic A. D. Luthans F. (1998). Social cognitive theory and self-efficacy: Goin beyond traditional motivational and behavioral approaches. Organ. Dyn. 26, 62–74. doi: 10.1016/S0090-2616(98)90006-7

[ref99] Stiglbauer B. Gnambs T. Gamsjäger M. Batinic B. (2013). The upward spiral of adolescents' positive school experiences and happiness: investigating reciprocal effects over time. J. Sch. Psychol. 51, 231–242. doi: 10.1016/j.jsp.2012.12.002, PMID: 23481087

[ref100] Sutton R. E. Harper E. (2009). “Teachers' emotion regulation” in International Handbook of Research on Teachers and Teaching. eds. Saha L. J. Dworkin L. G. (New York: Springer), 389–402.

[ref101] Sutton R. E. Mudrey-Camino R. Knight C. C. (2009). Teachers' emotion regulation and classroom management. Theory Pract. 48, 130–137. doi: 10.1080/00405840902776418

[ref102] Sweetman D. Luthans F. (2010). “The power of positive psychology: psychosocial capital and work engagement” in Work Engagement: A Handbook of Essential Theory and Research. eds. Bakker A. B. Leiter M. (New York, NY: Psychology Press), 54–68.

[ref103] Tabachnick B. G. Fidell L. S. (2007). Using Multivariate Statistics. 5th. Boston, MA: Pearson Education.

[ref104] Talbot K. Mercer S. (2018). Exploring university ESL/EFL teachers’ emotional well-being and emotional regulation in the United States, Japan and Austria. Chin. J. Appl. Linguist. 41, 410–432. doi: 10.1515/cjal-2018-0031

[ref105] Tang Y. He W. (2022). Emotion regulation and psychological capital of Chinese university students during the Covid-19 pandemic: the serial mediation effect of learning satisfaction and learning engagement. Int. J. Environ. Res. Public Health 19:13661. doi: 10.3390/ijerph192013661, PMID: 36294240 PMC9603398

[ref106] Thompson R. A. (1994). Emotion regulation: a theme in search of definition. Monogr. Soc. Res. Child Dev. 59, 25–52. doi: 10.1111/j.1540-5834.1994.tb01276.x, PMID: 7984164

[ref107] Tosten R. Toprak M. (2017). Positive psychological capital and emotional labor: a study in educational organizations. Cogent Educ. 4:1301012. doi: 10.1080/2331186X.2017.1301012

[ref108] Tugade M. M. Fredrickson B. L. (2007). Regulation of positive emotions: emotion regulation strategies that promote resilience. J. Happiness Stud. 8, 311–333. doi: 10.1007/s10902-006-9015-4

[ref109] Viseu J. de Jesus S. N. Rus C. Canavarro J. M. (2016). Teacher motivation, work satisfaction, and positive psychological capital: a literature review. Electron. J. Res. Educ. Psychol. 14, 439–461. doi: 10.25115/ejrep.39.15102

[ref110] Xiao Y. Fathi J. Mohammaddokht F. (2022). Exploring a structural model of teaching enjoyment, teacher self-efficacy, and work engagement. Front. Psychol. 13:918488. doi: 10.3389/fpsyg.2022.918488, PMID: 35783707 PMC9244399

[ref111] Xie F. (2021). A study on Chinese EFL teachers' work engagement: the predictability power of emotion regulation and teacher resilience. Front. Psychol. 12:735969. doi: 10.3389/fpsyg.2021.735969 34512487 PMC8430242

[ref112] Zhang L. J. Saeedian A. Fathi J. (2022). Testing a model of growth mindset, ideal L2 self, boredom, and WTC in an EFL context. J. Multiling. Multicult. Dev., 1–16. doi: 10.1080/01434632.2022.2100893

[ref113] Zhang Y. Zhang S. Hua W. (2019). The impact of psychological capital and occupational stress on teacher burnout: mediating role of coping styles. Asia Pac. Educ. Res. 28, 339–349. doi: 10.1007/s40299-019-00446-4

[ref114] Zheng S. Heydarnejad T. Aberash A. (2022). Modeling the interplay between emotion regulation, self-efficacy, and L2 grit in higher education. Front. Psychol. 13:1013370. doi: 10.3389/fpsyg.2022.1013370, PMID: 36211869 PMC9536314

[ref115] Zimmerman B. J. Bandura A. Martinez-Pons M. (1992). Self-motivation for academic attainment: the role of self-efficacy beliefs and personal goal setting. Am. Educ. Res. J. 29, 663–676. doi: 10.3102/00028312029003663

